# Does the essential oil of *Lippia sidoides* Cham. (pepper-rosmarin) affect its endophytic microbial community?

**DOI:** 10.1186/1471-2180-13-29

**Published:** 2013-02-07

**Authors:** Thais Freitas da Silva, Renata Estebanez Vollú, Diogo Jurelevicius, Daniela Sales Alviano, Celuta Sales Alviano, Arie Fitzgerald Blank, Lucy Seldin

**Affiliations:** 1 Instituto de Microbiologia Paulo de Góes, Universidade Federal do Rio de Janeiro, Centro de Ciências da Saúde, Bloco I, Ilha do Fundão, Rio de Janeiro CEP 21941-590, Brazil; 2 Departamento de Engenharia Agronômica, Universidade Federal de Sergipe, Aracajú, SE CEP 49100-000, Brazil

**Keywords:** *Lippia sidoides*, Essential oil, Stem, Leaf, Endophytic bacteria and fungi, Plant-microorganism interaction

## Abstract

**Background:**

*Lippia sidoides* Cham., also known as pepper-rosmarin, produces an essential oil in its leaves that is currently used by the pharmaceutical, perfumery and cosmetic industries for its antimicrobial and aromatic properties. Because of the antimicrobial compounds (mainly thymol and carvacrol) found in the essential oil, we believe that the endophytic microorganisms found in *L. sidoides* are selected to live in different parts of the plant.

**Results:**

In this study, the endophytic microbial communities from the stems and leaves of four *L. sidoides* genotypes were determined using cultivation-dependent and cultivation-independent approaches. In total, 145 endophytic bacterial strains were isolated and further grouped using either ERIC-PCR or BOX-PCR, resulting in 76 groups composed of different genera predominantly belonging to the Gammaproteobacteria. The endophytic microbial diversity was also analyzed by PCR-DGGE using 16S rRNA-based universal and group-specific primers for total bacteria, Alphaproteobacteria, Betaproteobacteria and Actinobacteria and 18S rRNA-based primers for fungi. PCR-DGGE profile analysis and principal component analysis showed that the total bacteria, Alphaproteobacteria, Betaproteobacteria and fungi were influenced not only by the location within the plant (leaf vs. stem) but also by the presence of the main components of the *L. sidoides* essential oil (thymol and/or carvacrol) in the leaves. However, the same could not be observed within the Actinobacteria.

**Conclusion:**

The data presented here are the first step to begin shedding light on the impact of the essential oil in the endophytic microorganisms in pepper-rosmarin.

## Background

*Lippia sidoides* Cham*.*, popularly known as pepper-rosmarin, is an aromatic and medicinal plant species of the family Verbenaceae. This plant is a typical shrub commonly found in northeast Brazil that produces a highly scented essential oil in its leaves. The *L. sidoides* essential oil has potential economic value because of its industrial use in the commercial production of perfumes, creams, lotions and deodorants [1]. Moreover, the leaves of *L. sidoides* are also extensively used in folk medicine for the treatment of acne, wounds, skin and scalp infections [1], allergic rhinitis and vaginal, mouth and throat infections [2]. When tested against different pathogenic bacteria, including *Staphylococcus aureus* and *Pseudomonas aeruginosa*, as well as different fungi, including yeasts, dermatophytes and filamentous fungi, the essential oil from *L. sidoides* proved to be very promising as an antimicrobial compound [3,4]. Additionally, it has been previously demonstrated that the *L. sidoides* essential oil has insecticidal activity against the coleopteran *Tenebrio molitor*, larvicidal activity against *Aedes aegypti* linn and acaricidal activity against the two-spotted spider mite (*Tetranychus urticae* Koch) [5-7]. Thus, the essential oil produced by *L. sidoides* is of great interest and value because of its bactericidal, fungicidal, molluscicidal and larvicidal properties.

The major constituents of the essential oil of *L. sidoides* are thymol and carvacrol, which are responsible for the remarkable inhibitory activity against microorganisms [1,8,9]. However, the environmental conditions (such as soil type, the use of organic or mineral fertilizers, temperature, humidity and exposure to the sun and wind) where *L. sidoides* is cultivated may influence the chemical composition of the volatile oils [9,10]. Additionally, the amount of the essential oil components produced can vary depending on the plant genotype [11].

In other plants, the presence of intracellular bacteria found in association with the essential oil cells, such as the lysigen lacunae in vetiver root (*Chrysopogon zizanioides*), and the participation of bacteria in the biotransformation of essential oils have been previously demonstrated [12-14]. However, no evidence exists to suggest the participation of the endophytic microbial community in the transformation of the essential oil in *L. sidoides*, which appears to be associated with plant trichomes [15]. Here, we hypothesize that this community is influenced by the production of the volatile compounds of the essential oil in *L. sidoides* leaves. To the best of our knowledge, few studies concerning the microbial endophytic community associated with *L. sidoides* have been performed to date that specifically use the genotypes and environmental conditions of northeast Brazil. Thus, the microbial communities from the stems and leaves of four *L. sidoides* genotypes (LSID003, LSID006, LSID104 and LSID105), which show different amounts of carvacrol and thymol, were determined using cultivation-dependent and cultivation-independent approaches. We used 16S rRNA-based universal and group-specific primers for total bacteria, Alphaproteobacteria, Betaproteobacteria and Actinobacteria, as well as 18S rRNA-based primers for fungi, in combination with molecular (PCR-DGGE) and statistical (Principal Component Analysis - PCA) tools to evaluate whether the essential oil affects the endophytic microbial community in pepper-rosmarin.

## Methods

### Plants, sampling and experimental conditions

This study was conducted at the Experimental Farm “The Rural Campus of UFS”, located in São Cristóvão (geographical coordinates: latitude 11°00′ S and longitude 37° 12′ W) in northeast Brazil. The soil of this area is characterized as a red-yellow argisoil with the following chemical characteristics: pH – 5.4; organic matter – 21.1 g dm^-3^; P – 2.3 mg dm^-3^; K – 0.09 cmolc dm^-3^ (Mehlich 1); Ca + Mg – 2.70 cmolc dm^-3^; Al – 0.71 cmolc dm^-3^; S - SO_4_^2−^– 0.76 cmolc dm^-3^; Zn – 0.97 mg dm^-3^, Cu – 0.66 mg dm^-3^; Fe – 82.9 mg dm^-3^; and Mn – 2.76 mg dm^-3^. The seedlings were produced by utilizing approximately 15 cm-staked herbaceous offshoots. A mixture of washed coconut shell powder and washed sand (2:1) and 20 g l^-1^ of Biosafra® organomineral biofertilizer (3-12-6) were used as substrata for the rooted cuttings. Seedlings of approximately 20 cm were then taken to the field. The experimental plot consisted of rows with spaces of 1 m between the rows and 1 m between plants. The soil was first fertilized with 3 l per m^2^ of aged bovine manure and four *L. sidoides* genotypes (LSID003, LSID006, LSID0104 and LSID0105) showing differences in their origin and the composition of the essential oils produced were planted in each row. The chemical composition of the essential oil produced by each genotype has been previously described by Blank et al. [16] and is summarized in Table 1. Drip irrigation was conducted daily.

**Table 1:** **Genotypes of pepper-rosmarin (*Lippia sidoides *Cham.), their origins, and the major constituents and yield of their essential oils**

Major chemical constituents (%)*				
**Genotype**	**Origin**	**Thymol**	**Carvacrol**	**Oil yield (ml plant**^**-1**^**)**
LSID003	Mossoró - RN (05° 08′ 28.3’’ S; 37° 23′ 58.0’’ W)	70.9 – 90.8	0.2 – 0.0	5.79
LSID006	Tabuleiro do Norte - CE (05° 14′ 05.4’’ S; 38° 11′ 35.0’’ W)	66.4 – 81.1	0.4 – 0.3	4.95
LSID104	Poço Redondo - SE (09° 58′ 09.2’’ S; 37° 51′ 50.3’’ W)	7.5 – 8.2	45.3 – 56.1	2.83
LSID105	Poço Redondo - SE (09° 58′ 12.9’’ S; 37° 51′ 49.2’’ W)	69.6 – 79.3	0.2 – 0.2	1.71

* These values correspond to individual measures performed in two consecutive years [16].

Three plants of each *L. sidoides* genotype were harvested in the morning period with the plants in full flower, and 20 pieces of stems (approximately 30 cm in length) with leaves were sampled from each plant.

Stem and leaf samples were surface sterilized by rinsing with 70% ethanol for 2 min, 2.5% sodium hypochlorite for 5 min, 70% ethanol for 30 sec and then washing three times with sterile distilled water. Only the stem samples were subjected to UV light exposure for 5 min prior to the final water wash. To check the efficiency of the disinfection procedure, 100 μl of the water used in the last wash was plated onto Trypticase Soy Broth (TSB) agar-containing plates and incubated for 5 days at 32°C. Samples that were not contaminated according to the culture-dependent sterility test were cut into pieces of approximately 5 cm, 3 g of each stem and leaf samples were homogenized with 10 ml of sterile distilled water in a sterilized mortar and pestle and used for counting and isolation of endophytic bacterial strains and for DNA isolation.

### Counting, isolation and DNA extraction of endophytic bacterial strains

To determine the colony forming units per ml (CFU ml^-1^) in the stems and leaves of the different *L. sidoides* genotypes, each macerated sample (1 ml) obtained after disinfection was mixed with 9 ml of distilled water, and serial dilutions of these samples were plated onto TSB agar plates containing 1% nystatin (50 μg ml^-1^) and incubated for 5 days at 32°C. Colonies presenting different morphological characteristics in each plate used were selected for further purification. Bacterial cultures were stored at −80°C in TSB with 10% glycerol. All isolates were first divided based on their Gram staining characteristics. Genomic DNA was extracted from all bacterial strains using the protocol described by Pitcher et al. [17]. DNA preparations were separated by electrophoresis on an 0.8% agarose gel in 1X Tris/Borate/EDTA (TBE) buffer [18] and visualized to assess their integrity, then stored at 4°C prior to PCR amplification.

### BOX-PCR, ERIC-PCR and the molecular identification of selected bacterial strains

Amplification reactions using the primers BOXA1R [19] and ERIC1R and ERIC2F [20] were performed in the following mix: 1 μl (50–100 ng) of target DNA; 5 μl of 5X PCR buffer (Promega, RJ, Brazil); 2.5 mM (ERIC) or 3.75 mM (BOX) MgCl_2_; 0.5 mM dNTP; 0.4 μM and 1 μM of the primers ERIC1R - ERIC2F or BOXA1R, respectively; and 0.5 U (ERIC) or 1.25 U (BOX) of *Taq* polymerase in a 25 μl final volume. The cycle applied was 1 × [7 min at 95°C], 35 × [1 min at 94°C, 1 min at 52°C (with ERIC primers) or 53°C (with BOXA1R primer), 8 min at 65°C] and a final extension of 16 min at 65°C. Negative controls (without DNA) were run during all amplifications. Agarose gel electrophoresis of PCR products was performed using 1.4% agarose in 1X TBE buffer at 90 V for 4 h at room temperature. The BOX and ERIC-PCR results were collected into matrices indicating the presence or absence (scored as 1 or 0, respectively) of bands. Dendrograms were constructed using Dice similarity coefficients and the unweighted pair group method with arithmetic mean (UPGMA) through the BioNumerics software package (Applied Maths, Ghent, Belgium).

For molecular identification of the selected isolates, their 16S rRNA coding gene was amplified by PCR using the pair of universal primers pA and pH and the conditions described in Massol-Deya et al. [21]. The PCR products were then sequenced by Macrogen (South Korea). The partial 16S rRNA gene sequences (~800 bp) were identified using the BLAST-N tool (http://blast.ncbi.nlm.nih.gov/) on the National Center for Biotechnology Information (NCBI) website using the GenBank non-redundant database. A phylogenetic tree was constructed based on partial 16S rRNA gene sequences using the neighbor-joining method. MEGA 5.1 software was used to calculate Jukes-Cantor distances. Bootstrap analyses were performed with 1,000 repetitions, and only values higher than 50% are shown in the phylogenetic tree.

### Susceptibility of the bacterial isolates to the essential oil obtained from *L. sidoides* genotypes LSID006 and LSID104

The determination of the minimum inhibitory concentration (MIC) was performed using a serial dilution technique in 0.2 ml thin-walled 8 strip cap microtubes as recommended by CLSI M7-A4 for bacteria [22]. Bacterial isolates from the four genotypes were tested for susceptibility. The investigated essential oils containing contrasting amounts of thymol and carvacrol (Table 1) were diluted seven times using doubling dilution, from 4 to 0.03 mg ml^-1^, and 1 μl of each dilution was added to 189 μl TSB with 10 μl of the bacterial suspension (cells grown to a O.D. = 0.09 at 625 nm, then diluted 50X in TSB). The microtubes were incubated for 48 h at 32°C. Positive controls consisted of inoculated growth medium without the essential oil. The results were based on visual growth of bacterial strains, which was confirmed after the aseptic addition of 30 μl of resazurin to the tubes and further incubation at 32°C for 30 min. The MIC was defined as the minimum concentration of the essential oil resulting in complete growth inhibition [23]. A paired two-sample *t*-test was used to compare the growth range of the strains tested with different concentrations of both essential oils. P values of <0.05 were considered statistically significant.

### DNA extraction from stem and leaf samples

The total microbial community DNA was extracted directly from stem and leaf samples (0.5 g of each sample in triplicate) using the FastPrep Spin kit for soil DNA (BIO 101 Systems, CA, USA). DNA preparations were visualized after electrophoresis in a 0.8% agarose gel in 1X TBE buffer to assess their integrity and then stored at 4°C prior to PCR amplification.

### PCR amplification of 16S rRNA and 18S rRNA coding genes from stem and leaf samples for use in DGGE

Fragments of 16S rRNA and 18S rRNA genes were PCR amplified using DNA from stem and leaf samples and the primers listed in Table 2 under the conditions previously described for each pair of primers [24-30].

**Table 2:** **Universal bacterial primers and group-specific primers (based on 16S rRNA) and fungal primers (based on 18S rRNA) used for PCR amplification of *L. sidoides *stem and leaf DNA for DGGE evaluation**

**Communities**	**Primers**	**Reference**	** Sequences **^**a**^
Total bacteria	*U968/**L1401**	[26]	*5′ACCGCGAAGAACCTTAC3′/
			5′GCGTGTGTACAAGACCC3′
Total bacteria	**799F**/**1492R**	[29]	5′AACMGGATTAGATACCCKG3′/
	*U968/L1401	[26]	5′TACGGYTACCTTGTTACGACT3′
Alphaproteobacteria	**F203α**/L1401	[30]	5′CCGCATACGCCCTACGGGGGAAAGATTTAT3′
	*U968/L1401	[26]	
Betaproteobacteria	**F948β**/L1401	[30]	5′CGCACAAGCGGTGGATGA3′
	*U968/L1401	[26]	
Actinobacteria	**F243**/L1401	[27]	5′ GGATGAGCCCGCGGCCTA 3′
	*U968/L1401	[26]	
Fungi	**EF4**/**ITS4**	[28]	5′GGAAGGGRTGTATTTATTAG3′/
	*ITS1f/**ITS2**	[24]	5′ TCCTCCGCTTATTGATATGC3′
		[25]	*5′CTTGGTCATTTAGAGGAAGTAA3′/
		[24]	5′GCTGCGTTCTTCATCGATGC3′

^a^ The sequences correspond to the primers in bold.

* Primer with a 40 bp GC-clamp (5^′^- CGCCCGCCGCGCGCGGCGGGCGGGGCGGGGGCACGGGGGG –3^′^) attached.

### DGGE and statistical analysis

DGGEs were performed using a Bio-Rad DCode Universal Mutation Detection System (Bio-Rad Laboratories, Munich, Germany). PCR products (approximately 300 ng) were applied directly to 8% (w/v) polyacrylamide gels in 1X TAE buffer (40 mM Tris-acetate [pH 8.3] and 1 mM disodium EDTA) containing a denaturing gradient of urea and formamide varying from either 40 to 60% (total bacteria, Alphaproteobacteria, Betaproteobacteria and Actinobacteria) or 20 to 70% (fungal community). The gels were run for 16 h at 60°C and 65 V. After electrophoresis, the gels were stained for 30 min with SYBR Green I (Invitrogen - Molecular Probes, Eugene, OR, USA) and photographed under UV light using a STORM apparatus (Amersham Pharmacia Biotech, Munich, Germany). The dendrograms were constructed after image capture and analysis using the Dice correlation coefficient, and cluster analysis was performed by the unweighted pair group method with average linkages (UPGMA) using the BioNumerics software.

**Figure 1:**
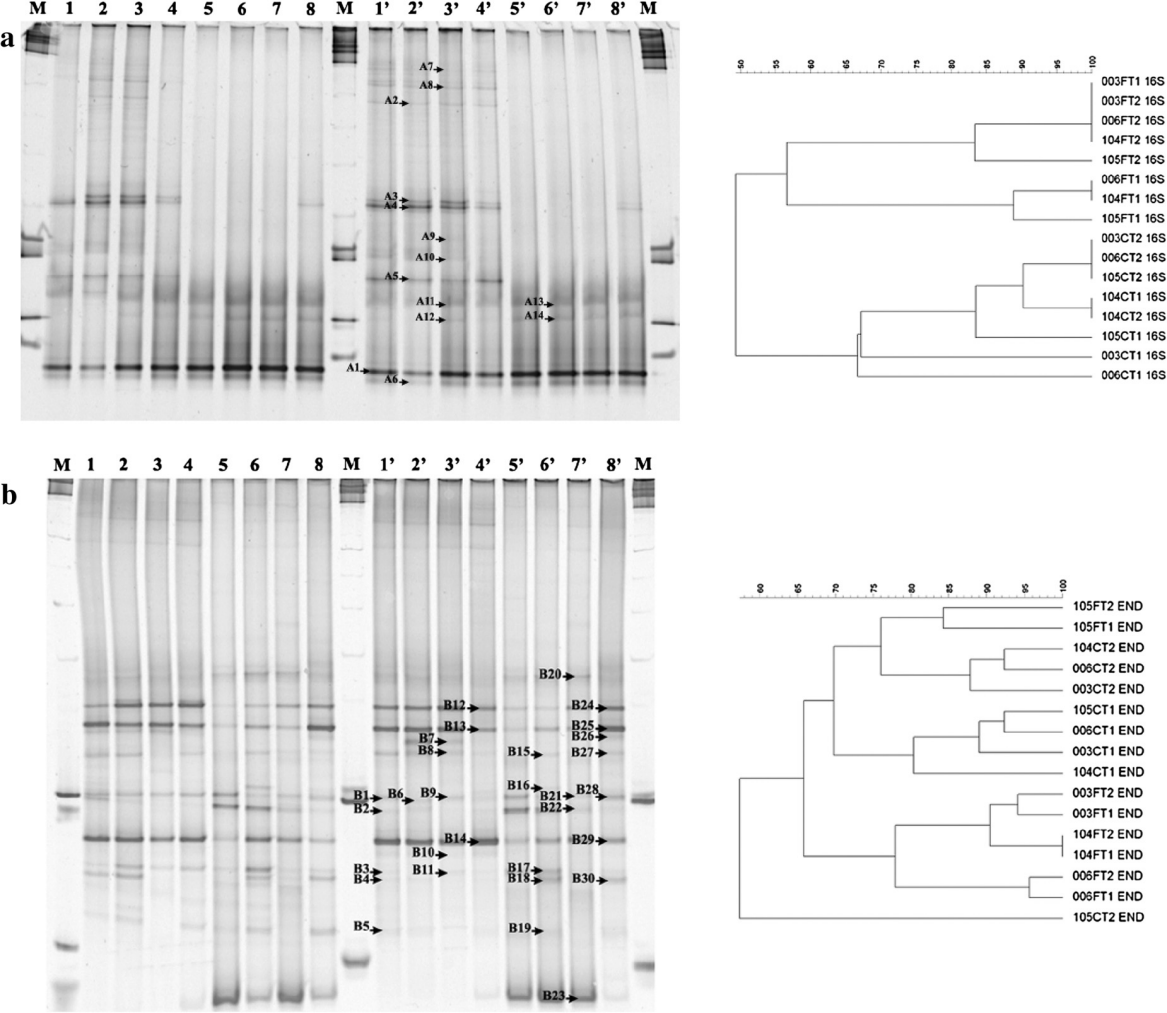
**Denaturing gradient gel electrophoresis (DGGE) fingerprints of bacterial 16S rRNA gene fragments amplified from stem and leaf DNA templates obtained from four genotypes of *Lippia sidoides *using the primers (a) U968/L1401 **[26]** and (b) 799F/1492R **[29]** followed by U968/L1401.** Two gels were used to compose this figure. Lanes 1, 2, 3, 4, 1′, 2′, 3′, 4′ – stem samples and 5, 6, 7, 8, 5′, 6′, 7′, 8′ – leaf samples from genotypes LSID003, LSID006, LSID104 and LSID105, respectively. Lanes marked with **M** correspond to a 1 kb ladder (Promega). Letters **A** and **B** followed by numbers indicate bands that were extracted from the gels **a** and **b**, respectively, for sequence analysis. The right side shows the corresponding dendrograms obtained after cluster analysis with mathematical averages (UPGMA) and Dice similarity coefficients comparing the total bacterial 16S rRNA gene fragments amplified from stem and leaf DNA templates obtained from four genotypes of *L. sidoides*. The genotypes are represented by the three first numbers (LSID - 003, 006, 104 and 105), followed by C or F for stem and leaf samples, respectively, and T1 and T2 corresponding to the replicates.

**Figure 2:**
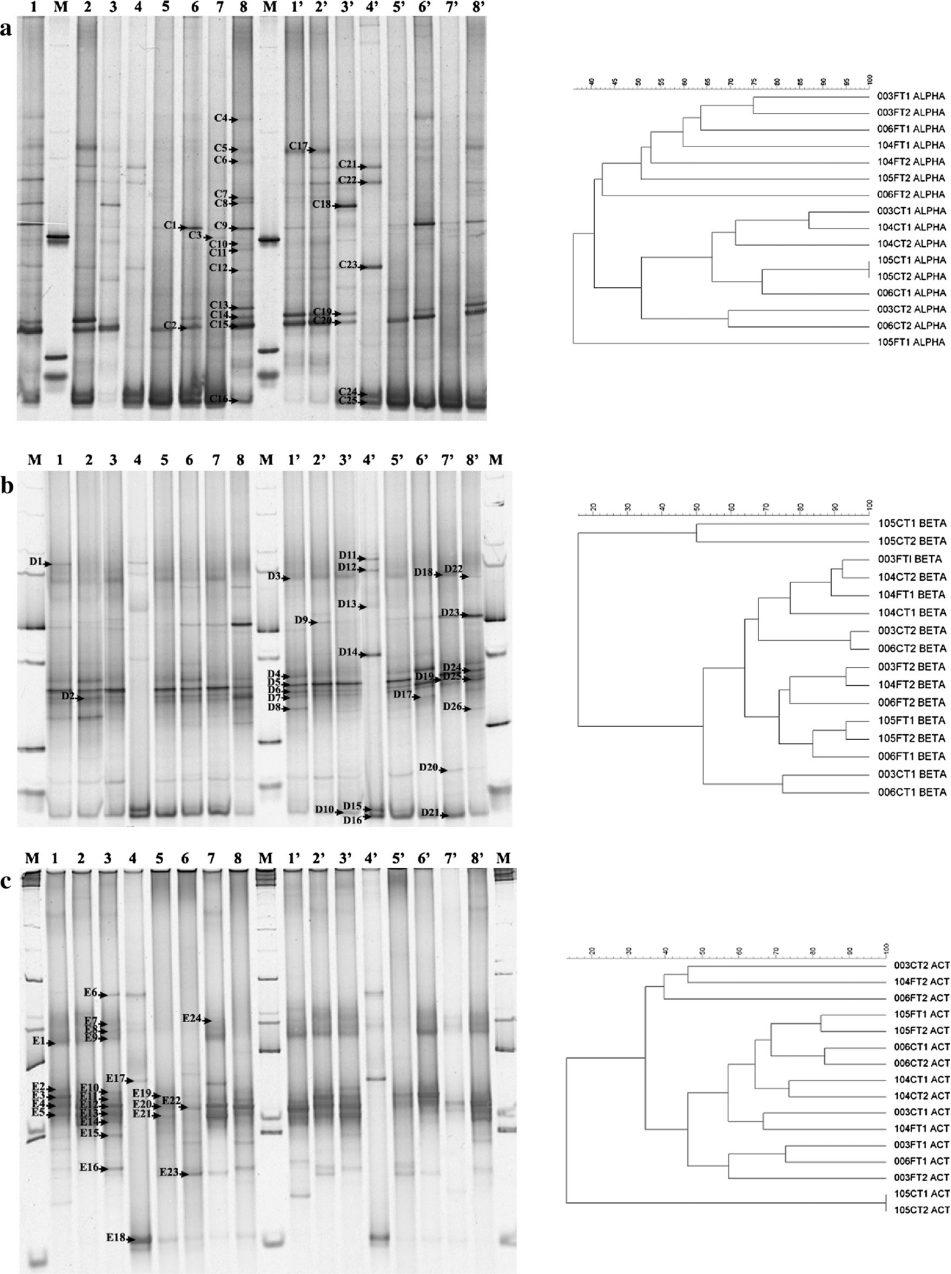
**Denaturing gradient gel electrophoresis (DGGE) fingerprints of bacterial 16S rRNA gene fragments amplified from stem and leaf DNA templates obtained from four genotypes of *Lippia sidoides *using the primers (a) F203α/L1401 and U968/L1401 **[26]**,**[30]** specific for Alphaproteobacteria, (b) F948β/L1401 and U968/L1401 **[26]**,**[30]** specific for Betaproteobacteria and (c) F243/L1401 and U968/L1401 **[26]**,**[27]** specific for Actinobacteria.** Two gels were used to compose figures (**a**), (**b**) and (**c**). Lanes 1, 2, 3, 4, 1′, 2′, 3′, 4′ – stem samples and 5, 6, 7, 8, 5′, 6′, 7′, 8′ – leaf samples from genotypes LSID003, LSID006, LSID104 and LSID105, respectively. Lanes marked with **M** correspond to a 1 kb ladder (Promega). Letters **C, D** and **E** followed by numbers indicate bands that were extracted from the gels **a**, **b** and **c**, respectively, for sequence analysis. The right side shows the corresponding dendrograms obtained after cluster analysis with mathematical averages (UPGMA) and Dice similarity coefficients comparing group-specific 16S rRNA gene fragments amplified from stem and leaf DNA templates obtained from four genotypes of *L. sidoides*. The genotypes are represented by the three first numbers (LSID - 003, 006, 104 and 105), followed by C or F for stem and leaf samples, respectively, and T1 and T2 corresponding to the replicates.

**Figure 3:**
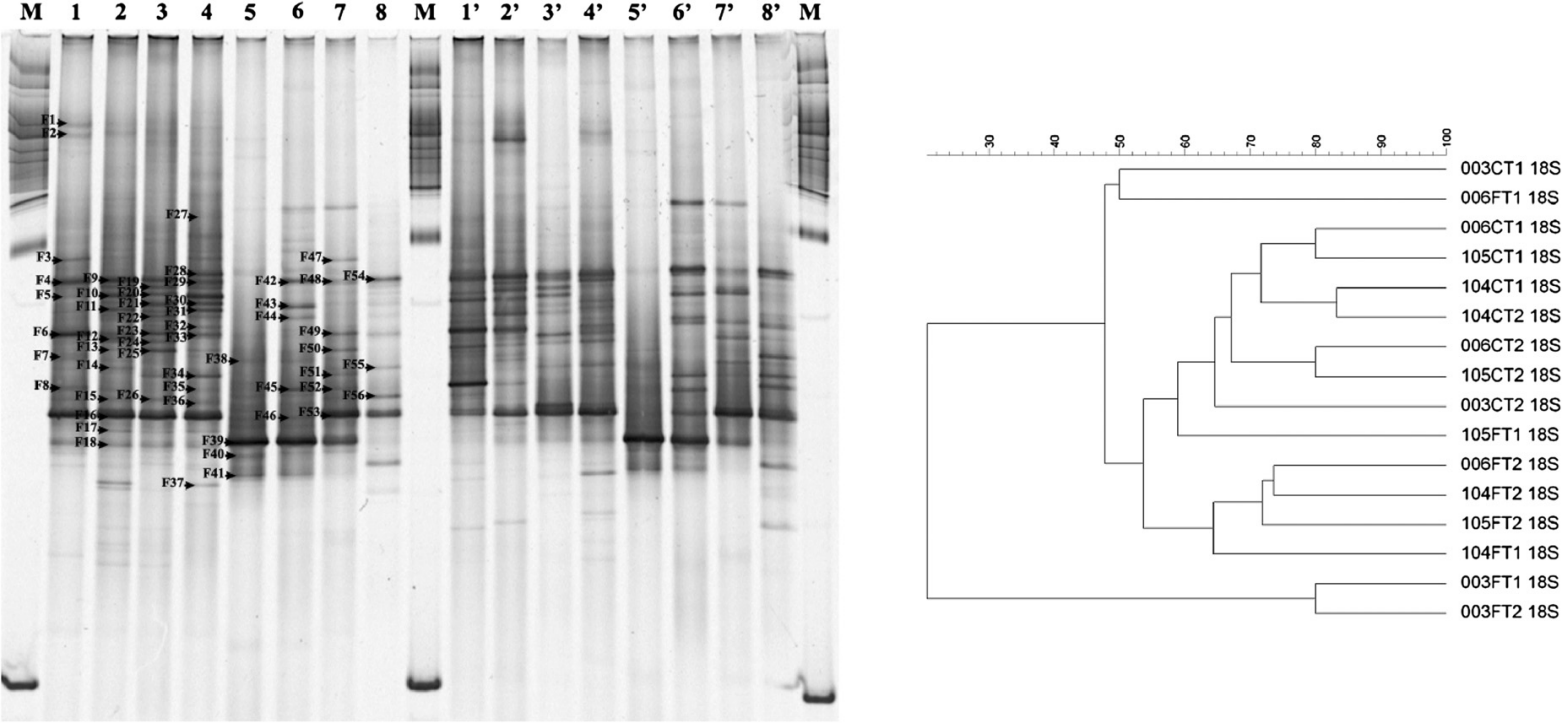
**Denaturing gradient gel electrophoresis (DGGE) fingerprints of fungal 18S rRNA gene fragments amplified from stem and leaf DNA templates obtained from four genotypes of *Lippia sidoides *using two sets of primers - EF4/ITS4 **[27]**,**[28]** and ITS1f/ITS2 **[24]**,**[25]**.** Lanes 1, 2, 3, 4, 1′, 2′, 3′, 4′ – stem samples and 5, 6, 7, 8, 5′, 6′, 7′, 8′ – leaf samples from genotypes LSID003, LSID006, LSID104 and LSID105, respectively. Lanes marked with **M** correspond to a 1 kb ladder (Promega). The letter **F** followed by numbers indicates bands that were extracted from the gels for sequence analysis. The right side shows the corresponding dendrogram obtained after cluster analysis with mathematical averages (UPGMA) and Dice similarity coefficients comparing the fungal 18S rRNA gene fragments amplified from stem and leaf DNA templates obtained from four genotypes of *L. sidoides*. The genotypes are represented by the three first numbers (LSID - 003, 006, 104 and 105), followed by C or F for stem and leaf samples, respectively, and T1 and T2 corresponding to the replicates.

Some bands were retrieved from the gels (marked in Figures 1, 2 and 3), reamplified as described above, and sequenced using each of the forward primers previously used (without a GC clamp). The partial 16S rRNA and 18S rRNA gene sequences were identified using the BLAST-N tool on the NCBI website and the GenBank non-redundant database.

The DGGE gels were analyzed to evaluate the distribution of the species and to correlate the profiles obtained with the *L. sidoides* essential oil constituents. Principal component analysis (PCA) was used as described previously [31] using the PC-ORD statistical software [32].

### Nucleotide sequence accession numbers

The nucleotide sequences determined in this study for the culturable bacterial community were deposited in the GenBank database under accession numbers JX471071 – JX471146 and for the DGGE band sequences in the DDBJ database under accession numbers AB778305 to AB778478.

## Results

### The bacterial community in the stems and leaves of four *L. sidoides* genotypes as determined by a cultivation-dependent approach

After disinfecting the stems and leaves of the different *L. sidoides* genotypes, serial dilutions of these samples were plated onto TSB agar plates for counting and selection of bacterial strains. Table 3 shows the determination of the colony forming units (CFU ml^-1^) in the stems and leaves. Across the four genotypes, the number of bacterial cells varied from zero to 1.6 × 10^3^ CFU ml^-1^ in the leaves and 1.2 to 3.4 x 10^5^ CFU ml^-1^ in the stems. Colonies presenting different morphologies in each plate used for counting were selected for further characterization. In total, 145 strains were collected: for stems, 37 were from LSID003, 36 from LSID006, 26 from LSID104 and 29 from LSID105; 17 strains were collected from the leaves of LSID105. The strains were then Gram-stained; 96 of the strains were Gram-negative and 49 were Gram-positive (Table 3). DNA from both Gram-negative and Gram-positive strains was then amplified using ERIC and BOX-PCR, respectively, for a preliminary screening of their diversity. Based on 70% similarity, 76 groups were formed: 49 groups originated from ERIC-PCR and 27 from BOX-PCR. While different groups were formed by a single strain, others were formed by two to six strains (data not shown).

**Table 3:** **Determination of the colony forming units per ml and characterization of the isolates in the stems and leaves of four *Lippia sidoides *genotypes**

	**STEMS**				**LEAVES**			
**Genotypes:**	**LSID003**	**LSID006**	**LSID104**	**LSID105**	**LSID003**	**LSID006**	**LSID104**	**LSID105**
CFU ml^-1^ (mean ± standard deviation)	1.2 ± 0.06 × 10^5 a^	3.4 ± 0.15 × 10^5 b^	1.2 ± 0.08 × 10^5 a^	2.6 ± 0.22 × 10^5 c^	0 ^d^	0 ^d^	0 ^d^	1.6 ± 0.4 × 10^3 e^
Number of isolates	37	36	26	29	0	0	0	17
Gram-positive (%)	24.3	22.2	69.2	0	0	0	0	82.5
Gram-negative (%)	75.7	77.8	30.8	100	0	0	0	17.7
Actinobacteria (%)	8.1	2.8	19.2	0	0	0	0	5.9
Firmicutes (%)	13.5	19.4	50	0	0	0	0	82.3
Gammaproteobacteria (%)	78.4	77.8	30.8	100	0	0	0	11.8

Values with the same letter are not statistically different based on the *t*-test at *p* = 0.05.

**Figure 4:**
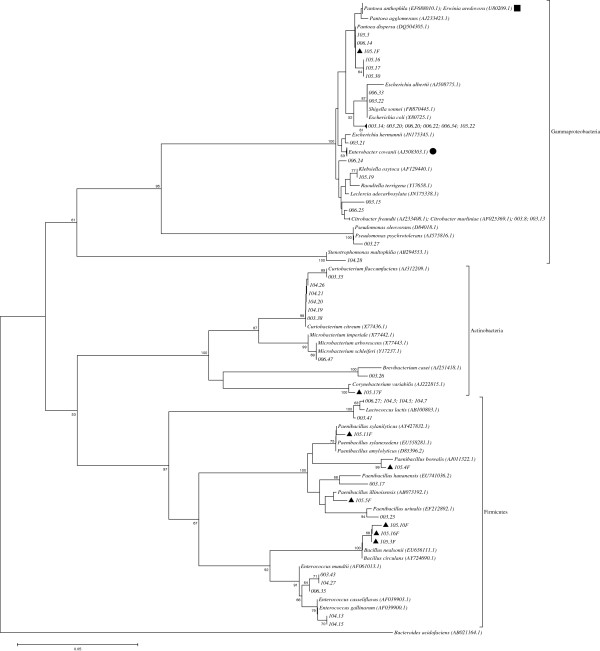
**Phylogenetic tree based on the 16S rRNA gene sequences (~800 pb) showing the relationship between the representative strains belonging to different BOX or ERIC groups with sequences of related species found by Blast searches.** The tree was constructed based on the neighbor-joining method. Bootstrap analyses were performed with 1000 repetitions and only values higher than 50 % are shown. The GenBank accession number of each bacterial species is enclosed in parentheses. The name of the isolated strains is formed by the different *Lippia sidoides* genotypes (LSID - 003, 006, 104 and 105), followed by a number. The number preceded by a black triangle and followed by the letter F corresponds to a strain isolated from the leaf samples, while without the triangle and the letter F from stem samples. The symbols (■) and (●) in the tree branches represent the isolates 003.1; 003.4; 003.5; 003.32; 003.34; 006.1; 006.2; 006.4; 006.7; 006.8; 006.10; 104.24; 105.1; 105.28 and 003.10; 003.12; 003.23; 003.24; 006.13; 006.16; 006.17; 006.18; 006.51; 104.10; 105.6; 105.12, respectively.

PCR fragments (~800 bp) obtained from part of the 16S rRNA coding gene of one representative strain belonging to different ERIC and BOX groups were sequenced, and the sequences obtained were compared to those in GenBank using the BLAST-N tool. Different genera could be associated with the sequences analyzed (Figure 4), with the majority of the strains (66.2%) being associated with Gammaproteobacteria and the remaining ones with Firmicutes and Actinobacteria. Strains isolated from the leaves were predominantly related to Firmicutes or Actinobacteria. While some genera/species were found exclusively in one genotype (for example: *Stenotrophomonas maltophila* was only found in the stems of LSID104 and *Pseudomonas psychrotolerans*, *Brevibacterium casei and Citrobacter freundii*/*C. murliniae* in LSID003), others could be detected in all genotypes, such as *Pantoea/Erwinia* and *Enterobacter cowanii*. Two other genera (*Bacillus* and *Corynebacterium*) were exclusively found in the leaves of LSID105 (Figure 4). The isolates found were associated with *B. nealsonii*/*B. circulans* and *C. variabilis*, respectively. The most diverse culturable endophytic bacterial community was observed within the stems of the LSID003 genotype, while the least diverse was found in the stems of LSID105 (Figure 4).

**Figure 5:**
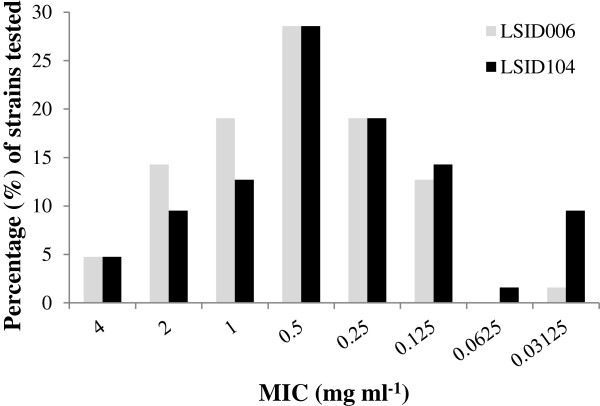
Minimum inhibitory concentration (MIC) determination of the isolated strains for the essential oil from genotypes LSID006 and LSID104.

To determine the susceptibility of the bacterial isolates to the essential oil obtained from *L. sidoides* genotypes LSID006 and LSID104 containing contrasting amounts of thymol and carvacrol (Table 1), MICs were determined by a doubling dilution technique using the two essential oils at eight concentrations (from 4 to 0.03 mg ml^-1^). From the MIC determination (Figure 5), 85.7% and 74.6% of the strains tested presented a MIC ≥ 0.25 mg ml^-1^ for the essential oil from genotypes LSID006 and LSID104, respectively, suggesting an intermediate susceptibility of the isolates to the presence of both essential oils. When a paired two-sample *t*-test was used, the strain susceptibility pattern against each of the essential oils was considered statistically significant (*P* = 0.05).

### The bacterial community in the stems and leaves of four *L. sidoides* genotypes as determined by a cultivation-independent approach

In a cultivation-independent approach (PCR-DGGE), the endophytic bacterial, actinobacterial and fungal communities were evaluated with respect to their structures in the stems and leaves of *L. sidoides* genotypes. Highly reproducible PCR-DGGE profiles were obtained from triplicate samples (stems and leaves from the four genotypes) from all communities evaluated in our experiment, indicating the robustness of the PCR-DGGE analyses (data not shown). To facilitate the comparison and further extraction of bands, two replicates per sample were loaded onto each gel.

The total bacterial community was first evaluated using the 16S rRNA primer pairs described by Nübel et al. [26]. The DGGE profiles were found to be very similar when DNA samples (stems or leaves) obtained from the four genotypes were compared. However, the same was not observed when the stem-derived samples were compared to leaf-derived samples (Figure 1a). Although certain common bands were detected in all of the samples, it appears that the colonization of the interior of the stems of *L. sidoides* is dominated by strains that are different from those found in the leaves. Cluster analysis corroborated the visual interpretation of the DGGE profiles, as stem-derived samples were separated from leaf-derived samples at approximately 50% (Figure 1a). Some bands (marked with the letter A, followed by a number) were retrieved from the gel, reamplified and sequenced. Phylogenetic comparison of 14 bands revealed seven sequences affiliated with *Enterobacter* sp. (A2-A4, A7-A10), one with *Pantoea* sp. (A5) and six with chloroplast DNA (A1, A6, A11-A14).

To minimize the annealing of the 16S rRNA-based primers with chloroplast DNA, a nested-PCR using the primers described by Chelius and Triplett [29] in the first round of amplification was chosen to re-evaluate the endophytic bacterial community in *L. sidoides*. An increase in the number of bands in the DGGE gel was observed, resulting in the sequencing of 30 bands (marked in Figure 1b with the letter B, followed by a number). Likewise, the diversity of genera also increased with the phylogenetic affiliation of the PCR fragments, and sequences related to *Pantoea* (B8, B10, B11, B13, B14, B29), *Pseudomonas* (B1, B3, B4, B9, B30), *Enterobacter* (B6, B20, B25, B28), *Erwinia* (B2, B12), *Cronobacter* (B26, B27), *Rhizobium* (B5), *Lactococcus* (B7), and *Escherichia* (B24) could be found. Similar to the identification of the bacterial isolates, members of the Gammaproteobacteria were predominant in the endophytic bacterial community found in *L. sidoides* when molecular techniques were used. However, the remaining eight bands analyzed in Figure 1b, predominantly found in the leaves, were related to chloroplast DNA. Moreover, from the cluster analysis, we observed that stem-derived and leaf-derived samples were separated into two groups (Figure 1b), as previously demonstrated when the primers U968 and L1401 were used in a single PCR amplification round. *L. sidoides* genotypes do not seem to influence the endophytic bacterial community as much as the location in the plant where this community is found (stem vs. leaf) does (Figure 1b).

Because the Gammaproteobacteria appeared to predominate inside the *L. sidoides* plants studied, which made it difficult to recover members of the bacterial community found in low numbers, primers for specific bacterial groups were used to detect Alphaproteobacteria, Betaproteobacteria and Actinobacteria. When the nested-PCR described in Gomes et al. [30] for detecting Alphaproteobacteria was used, a clear distinction between the leaf-derived profiles and those from the stems could be observed in DGGE (Figure 2a). Twenty-five bands were retrieved from the gel (marked in Figure 2a with the letter C, followed by a number), and the resulting sequencing allowed the identification of predominantly *Rhizobium* sp. (15 bands: C1, C4-C15, C17, C20). One sequence could be associated with *Balneimonas* (C18) and another with *Agrobacterium* (C19). Still, five selected bands were related to chloroplast DNA (C2, C3, C16, C24, C25). However, two sequences were affiliated with the genus *Cronobacter* (C21, C22) and one band with *Pantoea* (C23), both of which belong to the Gammaproteobacteria. In the dendrogram, profiles obtained from stems were separated from those obtained from leaf samples at 40% similarity (Figure 2a). Again, a more prominent influence of the location within the plant could be observed within the community of Alphaproteobacteria found inside the four genotypes of *L. sidoides*.

Endophytic Betaproteobacteria found in the leaves and the stems of *L. sidoides* were determined using the primers described by Gomes et al. [30]. DGGE profiles (Figure 2b) show that the location in the plant where the Betaproteobacteria community was found also influenced the structure of this community, although this observation is more evident within the leaf-derived community. Cluster analysis corroborated the visual interpretation of the DGGE profiles because leaf-derived samples formed a group at 74% (Figure 2b). Plants from the genotype LSID105 appeared to select for the Betaproteobacteria community present in their stems, as a separate group was formed in the dendrogram at less than 20%. Furthermore, some bands (marked with the letter D, followed by a number) were retrieved from the gel, reamplified and sequenced. Phylogenetic comparison of 26 bands revealed seven sequences affiliated with the genus *Ralstonia* (D3-D6, D8, D18, D19), four with *Acidovorax* (D22, D24-D26), three with *Massilia* (D2, D11, D17), two with *Burkholderia* (D9, D20) and one band related to each of the following genera: *Comamonas* (D23), *Cupriavidus* (D1), *Stenotrophomonas* (D7), *Enterobacter* (D12), *Cronobacter* (D14) and *Pantoea* (D15). Unexpectedly, the last four genera do not belong to the Betaproteobacteria, but rather to the Gammaproteobacteria which was the predominant class observed in total bacterial community inside the *L. sidoides* plants studied. Bands D10, D13, D16 and D21 were related to chloroplast DNA. While the genera *Comamonas* and *Acidovorax* were only found in leaf samples, *Cupriavidus* appears to be exclusive to stems.

For the structure characterization of Actinobacteria, the PCR amplification was performed as described in Heuer et al. [27]. DGGE profiles showed that the samples from either the leaves or the stems were less similar among the genotypes than for the other communities studied (Figure 2c). Based on the dendrogram, no specific groupings were observed. The location where the actinobacterial community was found (stem vs. leaf) does not seem to influence its structure. Similar to the Betaproteobacteria, plants from the genotype LSID105 may have selected the actinobacterial community in their stems because a separate group was formed in the dendrogram at less than 15% (Figure 2c).

Twenty-four bands were retrieved from the DGGE gel (marked in Figure 2c with the letter E, followed by a number). From the sequenced bands, 17 sequences could be associated with the genus *Microbacterium* (E1-E9, E11-E14, E19-E21, E24), two with Actinobacteria (E10, E22) and one sequence for each of the following genera: *Brachybacterium* (E15), *Cellulomonas* (E16) and *Nocardioides* (E23). Two bands were related to chloroplasts (E17, E18).

Although fungal communities were not evaluated by cultivation-dependent approaches, their diversity was determined in the stems and leaves of the four genotypes of *L. sidoides* by PCR-DGGE (using the primers listed in Table 2), contributing to a better understanding of the microbial communities associated with this plant. The resulting DGGE profiles (Figure 3) were more complex than the profiles obtained for the bacterial communities. However, an evident distinction between the leaf-derived profiles and those from the stems could be observed in DGGE, as it was observed for the total bacteria, Alphaproteobacteria and Betaproteobacteria. Two groups were formed at 54% in the resulting dendrogram based on the location in the plant (Figure 3). Plants from the genotype LSID003 seemed to select the fungal community present in their leaves, as a separate group was formed in the dendrogram at approximately 20%. Different bands were retrieved from the gel (marked in Figure 3 with the letter F, followed by a number), and their phylogenetic comparison revealed 29 sequences associated with the genus *Lasiodiplodia* (F2-F4, F6, F8-F10, F12, F13, F15-F18, F20, F21, F23-F26, F30-F35, F47, F50, F52, F53), 11 with *Botryosphaeria* (F1, F5, F7, F11, F14, F19, F22, F36, F48, F49, F51), seven with *Mycosphaerella* (F38-F40, F42, F43, F45, F46), two with *Corynespora* (F55, F56) and one with each of the following genera: *Neoaleurodiscus* (F27), *Ceratobasidium* (F29), *Heteroacanthella* (F37), *Pantospora* (F41), *Passalora* (F44) and *Massarinaceae* (F54). While bands related to the genera *Neoaleurodiscus* and *Heteroacanthella* were found in the stems, *Mycosphaerella*, *Pantospora*, *Passalora*, *Massarinaceae* and *Corynespora* were exclusively detected in the leaves. Although a few members of the Basidiomycota (*Ceratobasidium* and *Heteroacanthella*) were present, the majority of the bands from both leaves and stems were associated with the Ascomycota.

### Principal component analysis (PCA) of DGGE patterns

**Figure 6:**
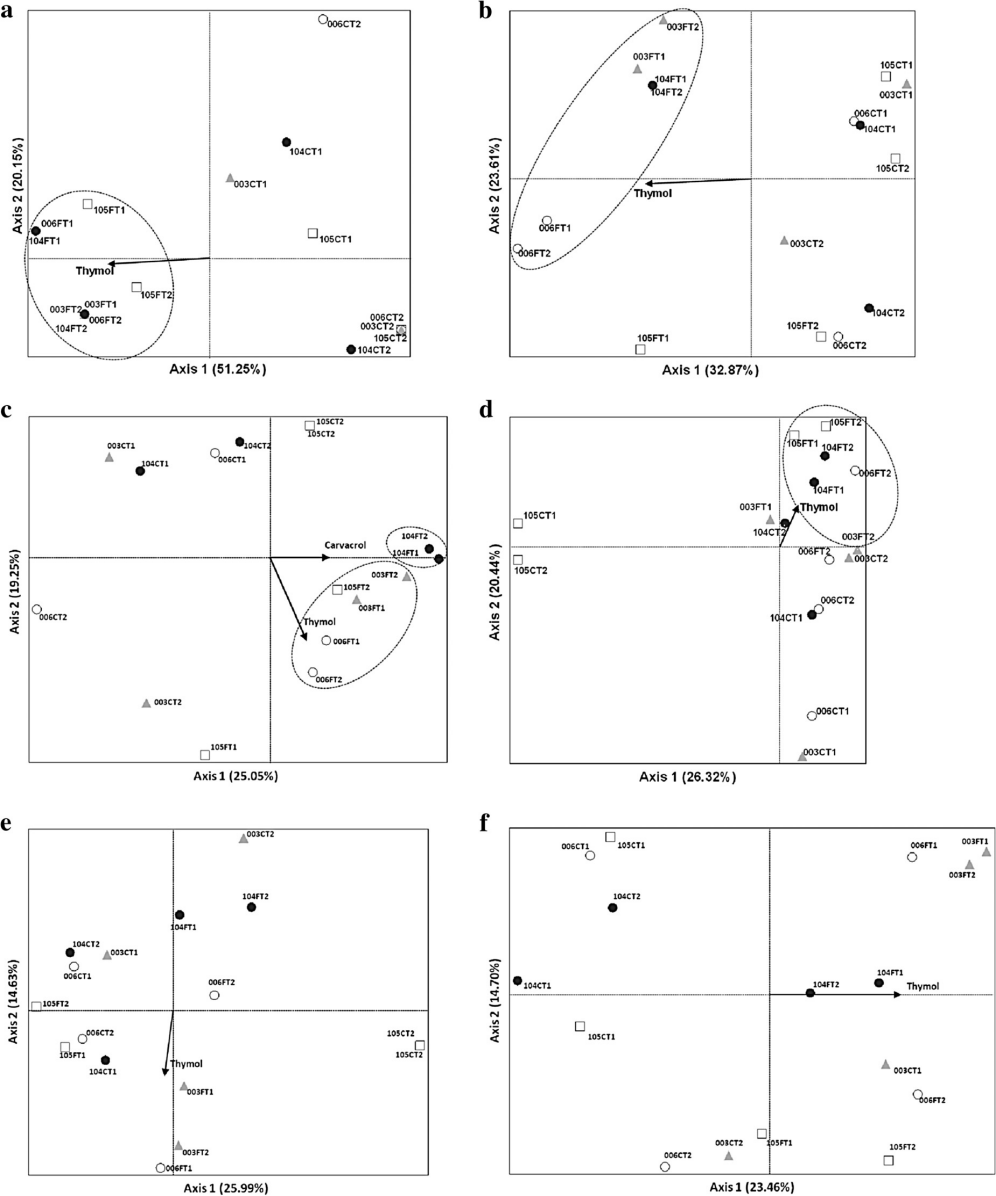
**Principal component analysis (PCA) ordination diagram with stem and leaf samples from *Lippia sidoides *genotypes LSID003, LSID006, LSID104 and LSID105 and the components of the essential oil (thymol and carvacrol) as variables (arrows): first axis - horizontal, second axis - vertical.** The fraction of the total variance accounted for by each axis is indicated in parentheses. The corresponding communities analyzed are as follows: (**a**) (**b**) total bacteria, (**c**) Alphaproteobacteria, (**d**) Betaproteobacteria, (**e**) Actinobacteria and (**f**) fungi. The genotypes are represented by the three first numbers (LSID - 003, 006, 104 and 105), followed by C or F for stem and leaf samples and T1 and T2 corresponding to the replicates.

Ordination of the PCR-DGGE profiles using PCA supported the aforementioned effects of plant location on the bacterial (Alphaproteobacteria and Betaproteobacteria) and fungal communities (Figure 6a, b, c, d, f). This effect was not clearly observed for the actinobacterial community (Figure 6e).

The first PCA axes explained 51.2, 32.8, 25.0, 26.3, 25.9 and 23.4% of the variance, whereas the second ones covered 20.1, 23.6, 19.2, 20.4, 14.6 and 14.7% (Figure 6a, b, c, d, e, f, respectively). With respect to the total bacterial communities, PCA ordination of the samples showed a tendency for these communities to group based on their origin, i.e., from the leaves or from the stems, mainly along the second axis (Figure 6a, b). Furthermore, it was possible to separate the leaf-derived samples in accordance to the presence of thymol (Figure 6a, b). PCA of the samples from the Alphaproteobacteria showed a separation along the first and second axes of the communities found in the leaves and in the stems (Figure 6c). While the leaf-derived samples belonging to the genotypes LSID003, LSID006 and LSID105 were grouped in accordance to the presence of thymol, those from LSID104 were also correlated with the presence of carvacrol (Figure 6c). Likewise, PCA of the Betaproteobacteria samples showed the tendency to group according to plant location. Stem-derived samples were separated from leaf-derived samples mainly along the first axis. The Betaproteobacteria community found in the leaves was also associated with the presence of thymol (Figure 6d). With respect to the Actinobacteria, PCA ordination of the samples did not show any tendency to group, along either the first or second axes (Figure 6e). In this case, the presence of thymol does not seem to be related to the actinobacterial communities found in the leaves of *L. sidoides* (Figure 6e). Finally, PCA ordination of the fungal communities showed a loose grouping in the function of the plant location along the second axis (Figure 6f). Again, the essential oil component, thymol, may have a positive effect on the selection of the leaf-derived fungal communities.

## Discussion

The interaction between plants and microorganisms has already been studied in different essential oil-producing plants, such as vetiver [13,14,33] and basil [34]. In a few cases, the microbial community associated with the plant interferes with the composition of the essential oil [13,14]. Thus far, there is no evidence that the essential oil produced in the leaves of *Lippia sidoides* (pepper-rosmarin), which is composed mainly of the two strongly antimicrobial monoterpenes thymol and carvacrol, is biotransformed inside the plant. Additionally, no data were available in the literature showing whether the essential oil interferes with the diversity of the microbial communities found inside of the plant and in strict contact with the volatile components of the essential oil. Therefore, we used cultivation-dependent and cultivation-independent methods to analyze microorganisms to increase our understanding of the behavior of the different microbial communities present in the stems and leaves (where the essential oil is found) of *L. sidoides*.

The CFUs were determined following the disinfection of the stems and leaves of four genotypes of *L. sidoides*. Bacterial colonization of the interior of *L. sidoides* was expected as it was previously observed in other plants [35,36]. However, no bacterial cells were recovered from the leaves of three genotypes (LSID003, LSID006 and LSID104), and the number of colonies from the leaves of the remaining genotype was much lower than the CFUs found in the stems. We hypothesize that the cells were killed when they were placed in direct contact with the essential oil after the maceration of the leaves. The endophytic bacteria found inside the stems would be better protected against the antimicrobial effect of the essential oil. To support this argument, the susceptibility of the bacterial isolates to the essential oil obtained from *L. sidoides* genotypes LSID006 and LSID104 was determined. The essential oil from the genotype LSID006 was chosen to represent the ones from LSID003 and LSID105 which are similar in their thymol and carvacrol contents. MIC determination showed that 85.7% and 74.6% of the strains tested presented a MIC ≥ 0.25 mg ml^-1^ of essential oil from genotypes LSID006 and LSID104, respectively, suggesting an intermediate sensitivity of the isolates to the presence of both essential oils. However, no difference in the susceptibility range could be observed between the stem-derived and leaf-derived strains. It is important to state that the number of leaf-derived strains tested was much lower than the number of stem-derived strains, thus compromising the interpretation of the results obtained.

In total, 145 endophytic bacterial isolates were obtained mostly from the stems. Our results suggest that the most dominant group associated with the *L. sidoides* genotypes was the Gammaproteobacteria, which is consistent with other studies [33,37,38]. Isolates from the genera *Bacillus* and *Paenibacillus* (belonging to the Firmicutes) were mainly obtained from LSID105 leaves (Figure 4). Because members of these genera are spore formers, they may have resisted exposure to the essential oil after maceration of the leaves. Although we do not know whether the isolated strains have any plant growth promoting potential, other studies have already demonstrated the importance of the different genera found here as nitrogen fixers, phosphate solubilizers and/or auxin producers in other plants [39,40].

As the cultivation-dependent methodology used was affected by cell death in the leaves, the PCR-DGGE approach chosen to determine the structure of the microbial communities found in the leaves and stems of *L. sidoides* became crucial to this study. Moreover, it allowed access to the communities (such as the Alphaproteobacteria, Betaproteobacteria and Actinobacteria) possibly present in lower numbers or that failed to grow under the conditions used for isolation.

Similar results were obtained when the total bacteria (accessed by two different sets of primers for PCR amplification), Alphaproteobacteria and Betaproteobacteria communities were considered. Slight differences in DGGE profiles were observed among the genotypes; nevertheless, these differences did not contribute to the grouping of the different communities as much as the location in the plant (stem or leaf) where these communities were found. In contrast, plant genotypes were shown to have great influence on the microbial communities associated with other plants [41,42].

While total bacteria and Betaproteobacteria were correlated with the presence of thymol in the leaves, the Alphaproteobacteria community was correlated with the presence of both thymol and carvacrol (more specifically in the genotype LSID104 where carvacrol is the main essential oil component). Because *Rhizobium* was the predominant genus detected within the Alphaproteobacteria community, we may assume that it can withstand the presence of the volatile components of the essential oil. The same postulation can be made for the genera *Comamonas* and *Acidovorax* because they were only found in samples from leaves. In contrast, no specific grouping was observed when Actinobacteria were considered. Actinobacterial communities do not seem to be influenced drastically by plant location or the presence of the essential oil in the leaves of *L. sidoides*. It is well documented that Actinobacteria are particularly adapted to survival in harsh environments [43], which may explain why strains belonging to the genera *Curtobacterium*, *Microbacterium*, *Brevibacterium* and *Corynebacterium* were isolated in this study. *Corynebacterium* was the only actinobacterial genus found in the leaves (genotype LSID105).

When the fungal communities were evaluated, we also observed the influence of the part of the plant sampled on their structure, as previously demonstrated for bacteria. However, the DGGE profiles were more complex, and a greater diversity of genera was observed within the fungal communities. The phylum Ascomycota was prevalent among the different fungal taxa found. Similarly, Siqueira et al. [44] isolated endophytic fungi representing different species belonging to the groups Ascomycota, Coelomycetes and Hyphomycetes from *L. sidoides* Cham. In *Hevea brasiliensis* (rubber tree), Gazis and Chaverri [45] observed fungal communities present in the leaves that were different from those isolated from the stem. Ascomycota was also the prevalent fungal group found. Based on PCA, fungal communities were to some extent correlated with the presence of thymol in the leaves.

## Conclusion

On the basis of the data from bacterial and fungal communities found in the leaves and stems of different genotypes of *L. sidoides*, we believe that both communities are selected by the conditions found in the interior of the plant. Thus, the presence of an essential oil with antimicrobial properties in the leaves certainly represents harsh survival conditions for the endophytic microorganisms. To understand how the microbial community associated with *L. sidoides* contributes to the physiology of the plant is the next step to be achieved.

## Competing interests

The authors declare that they have no competing interests.

## Authors’ contributions

TFS, REV and DJ carried out the experiments and LS wrote the manuscript. DSA, CSA and AFB made significant contribution on *Lippia sidoides* physiology and cultivation. All of the authors examined and agreed with the final manuscript.
